# Reach and Use of Diabetes Prevention Services in the United States, 2016-2017

**DOI:** 10.1001/jamanetworkopen.2019.3160

**Published:** 2019-05-10

**Authors:** Mohammed K. Ali, Kai McKeever Bullard, Giuseppina Imperatore, Stephen R. Benoit, Deborah B. Rolka, Ann L. Albright, Edward W. Gregg

**Affiliations:** 1 Hubert Department of Global Health, Rollins School of Public Health, Emory University, Atlanta, Georgia; 2 Department of Family and Preventive Medicine, School of Medicine, Emory University, Atlanta, Georgia; 3 Division of Diabetes Translation, National Center for Chronic Disease Prevention and Health Promotion, Centers for Disease Control and Prevention, Atlanta, Georgia

## Abstract

**Importance:**

Coordinated efforts by national organizations in the United States to implement evidence-based lifestyle modification programs are under way to reduce type 2 diabetes (hereinafter referred to as diabetes) and cardiovascular risks.

**Objective:**

To provide a status report on the reach and use of diabetes prevention services nationally.

**Design, Setting, and Participants:**

This nationally representative, population-based cross-sectional analysis of 2016 and 2017 National Health Interview Survey data was conducted from August 3, 2017, through November 15, 2018. Nonpregnant, noninstitutionalized, civilian respondents 18 years or older at high risk for diabetes, defined as those with no self-reported diabetes diagnosis but with diagnosed prediabetes or an elevated American Diabetes Association (ADA) risk score (>5), were included in the analysis. Analyses were conducted for adults with (and in sensitivity analyses, for those without) elevated body mass index.

**Main Outcomes and Measures:**

Absolute numbers and proportions of adults at high risk with elevated body mass index receiving advice about diet, physical activity guidance, referral to weight loss programs, referral to diabetes prevention programs, or any of these, and those affirming engagement in each (or any) activity in the past year were estimated. To identify where gaps exist, a prevention continuum diagram plotted existing vs desired goal achievement. Variation in risk-reducing activities by age, sex, race/ethnicity, educational attainment, insurance status, history of gestational diabetes mellitus, hypertension, or body mass index was also examined.

**Results:**

This analysis included 50 912 respondents (representing 223.0 million adults nationally) 18 years or older (mean [SE] age, 46.1 [0.2] years; 48.1% [0.3%] male) with complete data and no self-reported diabetes diagnosis by their health care professional. Of the represented population, 36.0% (80.0 million) had either a physician diagnosis of prediabetes (17.9 million), an elevated ADA risk score (73.3 million), or both (11.3 million). Among those with diagnosed prediabetes, 73.5% (95% CI, 71.6%-75.3%) reported receiving advice and/or referrals for diabetes risk reduction from their health care professional, and, of those, 35.0% (95% CI, 30.5%-39.8%) to 75.8% (95% CI, 73.2%-78.3%) reported engaging in the respective activity or program in the past year. Half of adults with elevated ADA risk scores but no diagnosed prediabetes (50.6%; 95% CI, 49.5%-51.8%) reported receiving risk-reduction advice and/or referral, of whom 33.5% (95% CI, 30.1%-37.0%) to 75.2% (95% CI, 73.4%-76.9%) reported engaging in activities and/or programs. Participation in diabetes prevention programs was exceedingly low. Advice from a health care professional, age range from 45 to 64 years, higher educational attainment, health insurance status, gestational diabetes mellitus, hypertension, and obesity were associated with higher engagement in risk-reducing activities and/or programs.

**Conclusions and Relevance:**

Among adults at high risk for diabetes, major gaps in receiving advice and/or referrals and engaging in diabetes risk-reduction activities and/or programs were noted. These results suggest that risk perception, health care professional referral and communication, and insurance coverage may be key levers to increase risk-reducing behaviors in US adults. These findings provide a benchmark from which to monitor future program availability and coverage, identification of prediabetes, and referral to and retention in programs.

## Key Points

**Question:**

What number and proportion of US adults are referred to and use diabetes preventive services?

**Findings:**

In this nationally representative cross-sectional analysis of 50 912 survey respondents, 73.5% of those with diagnosed prediabetes and 50.6% of those with risk factors reported receiving any advice or referrals to reduce risk; of those advised, 35.0% to 75.8% of those with diagnosed prediabetes and 33.5% to 75.2% with risk factors reported engaging in various risk-reducing activities or programs in the past year. Those with a formal prediabetes diagnosis were more likely to receive advice or referrals by health care professionals, and those advised were more likely to engage in risk-reducing activities and/or programs.

**Meaning:**

To improve reach and use of diabetes preventive services, expansion of some combination of programs (supply), awareness (demand), and access and referrals by health care professionals (linkage) appears to be needed.

## Teaser

This nationally representative cross-sectional analysis of data from the US National Health Interview Survey findings assesses the reach and use of diabetes prevention services among US adults with diagnosed prediabetes or risk factors for type 2 diabetes.

## Introduction

A number of national organizations in the United States are currently coordinating efforts to deliver type 2 diabetes (hereinafter referred to as *diabetes*) prevention services to attempt to curb the economic and disabling physical and psychosocial effects of the disease.^1,2^ This effort is supported by robust evidence from the Diabetes Prevention Program (DPP) study and others,^3,4,5^ which showed that lifestyle modification (LSM) and/or insulin-sensitizing medications among people at high risk for diabetes can delay progression to diabetes onset. Behavioral counseling for LSM (to eat fiber-rich foods, reduce calories, be physically active, and manage weight) offers the most sustainable,^6,7^ most cost-effective,^8^ and broadest benefits in terms of lowering cardiovascular risks,^9^ disability, obstructive sleep apneas, retinopathy, and urinary incontinence.^10^ Accordingly, the US Preventive Services Task Force and American Diabetes Association (ADA) recommend screening and multiple-visit lifestyle counseling to achieve weight loss and reduce diabetes risk in high-risk adults.^11,12^ To facilitate scaling LSM programs, several interventions applying principles from the DPP trial have been tested in community, workplace, health care, and online settings and have been associated with at least modest benefits.^13,14,15^ In addition, commercial and public payers in the United States, including Medicare, now pay for LSM programs delivered to people at high risk for diabetes.

Coverage for diabetes prevention services is based on data and aspirations that these programs will be high-value investments by lowering long-term costs of medications, use of health care services, and lost productivity associated with diabetes,^16,17^ which currently total $327 billion annually.^1^ Notably, models suggest that the value from diabetes-prevention LSM programs is sensitive to enrollment by those at risk of diabetes, risk level, attendance at program sessions, and achievement of lifestyle goals.^5,13,18^ Also, awareness and internalization of one’s risk are crucial motivators to engage in and maintain behavioral changes. Previous data^2^ have shown that 88.4% of people with biochemically confirmed prediabetes (ie, those with elevated blood glucose levels not yet in the diabetes range) were unaware of their prediabetes.

Organized efforts to increase the availability of evidence-based programs online and in communities, health care settings, and workplaces have been aided by support and referral from clinical settings. Despite the initiation and momentum of these programs, few population-based data exist on their reach, implementation, and adoption. We herein assessed national progress in diabetes prevention and present findings using a prevention continuum diagram that facilitates identification of the most prominent gaps.

## Methods

### Data Source

The National Center for Health Statistics of the Centers for Disease Control and Prevention conducts the annual National Health Interview Survey (NHIS), the largest nationally representative cross-sectional survey of noninstitutionalized US civilians. The National Center for Health Statistics Research Ethics Review Board approved data collection in the NHIS. All participants provided informed oral consent before participation, and all data were deidentified before analyses. This analysis was exempted from review by the National Center for Health Statistics because we used only publicly available, deidentified data. This study followed the Strengthening the Reporting of Observational Studies in Epidemiology (STROBE) reporting guideline for cross-sectional studies.

### Data Collection

Details of the methods, survey instruments, data collection procedures, and analysis protocols of the NHIS are available online.^19^ Trained interviewers administered questionnaires to obtain social, demographic, economic, and behavioral characteristics of respondents. Respondents also self-reported their medical history, including information about medications, height, and weight.

The response rate for the 2016 NHIS was 67.9%^20^; for the 2017 NHIS, 66.5%.^21^ Approximately 4% of respondents had missing data on preventive health behaviors, family history, receipt of glucose tests, prediabetes diagnosis, or physical activity and were excluded from analyses. Our analyses included 50 912 nonpregnant, noninstitutionalized, civilian respondents 18 years or older with complete data and no self-reported diabetes diagnosis by their health care professional, representing 223.0 million US adults in 2017. Of these, 33 078 adults (representing 65.3% or 145.5 million) were overweight or obese. Analyses of the 2016 and 2017 NHIS Diabetes supplement were conducted from August 3, 2017 through November 15, 2018.

### Study Population

We estimated proportions of respondents considered to be at high risk for diabetes using several definitions. Among those without self-reported diabetes, we classified diagnosed prediabetes as a positive response to the question, “Other than during pregnancy, have you ever been told by a physician or other health professional that you have borderline diabetes or prediabetes?” For those at high risk without a known prediabetes diagnosis (ie, those with a negative response to the previous question), we used the ADA composite score^22^ of diabetes risk factors (age, sex, race/ethnicity, family history of diabetes, body mass index [BMI; calculated as weight in kilograms divided by height in meters squared], history of gestational diabetes mellitus [GDM], diagnosed hypertension, and lack of physical activity) (eTable 1 in the Supplement), which is highly sensitive for diabetes.^23^ A score of 5 or higher (possible range, 0-11) was considered high risk for diabetes. We also reported the prevalence and numbers with a high ADA risk score with diagnosed prediabetes.

### Statistical Analysis

We used SAS, version 9.3 (SAS Institute Inc) and SUDAAN, version 11.0 (Research Triangle Institute) software to account for the complex survey design. For all analyses, we calculated weighted percentages and standard errors or 95% CIs. To estimate the population size of those affected, we applied the weighted percentages to the July 1, 2017, US resident civilian and noninstitutionalized population estimates from the US Census Bureau.

We described the sociodemographic and clinical characteristics of each group. To assess the association of a prediabetes diagnosis with the actions of a health care professional and the likelihood of engagement in diabetes prevention activities, we stratified all analyses a priori by prediabetes diagnosis or elevated ADA risk score without a formal prediabetes diagnosis.

Because weight loss is 1 of the key LSM goals in diabetes prevention programs, we restricted analyses to overweight individuals (BMI≥23.0 for Asian American respondents or ≥25.0 for all others), which also aligns with eligibility criteria for the National Diabetes Prevention Program.^24^ We conducted sensitivity analyses to assess whether the addition of people with normal weight at high risk of diabetes changed our results.

We generated national diabetes prevention continuum diagrams—a tool used in health services research^25^—to assist with identification of gaps in delivery and uptake. To track how commonly health care professionals advised adults at high risk for diabetes to increase physical activity, reduce dietary fat or total calorie intake, or participate in weight loss programs or referred them to diabetes prevention programs in the past year, we calculated frequencies based on participant responses to survey questions (eTable 2 in the Supplement). Because variation often occurs in practice of what health care professionals choose or remember to emphasize, we also estimated what proportion of respondents reported receiving advice from their health care professionals for any of these activities. To assess engagement, we estimated proportions of respondents reporting that they reduced fat or total caloric intake, increased physical activity, participated in a weight loss program, or participated in a DPP-like intervention in the past year. Recognizing user variation in preferences and competing priorities, we conducted analyses to estimate proportions of respondents who engaged in any of the activities or programs in the past year, but we also examined each activity and program separately in sensitivity analyses.

We also examined whether engagement in any behavior to reduce diabetes risk, stratified by whether the participant was advised and/or referred by a health care professional, varied by year, age, sex, race/ethnicity, educational attainment, insurance status, history of GDM, hypertension, or BMI. To assess whether differences between groups were statistically significant, we used multivariate logistic regression models and presented adjusted prevalence estimates, using Wald *F* tests to assess whether associations were statistically significant. *P* < .05 was considered statistically significant for 2-sided tests. Unless otherwise indicated, data are expressed as percentages (SEs).

## Results

This analysis included 50 912 individuals, representing 223.0 million US adults without diabetes in 2017. The weighted study population (eTable 3 in the Supplement) had a mean (SE) age of 46.1 (0.2) years; 48.1% (0.3%) were male and 59.1% (0.3%) were female. For race/ethnicity, 65.1% (0.8%) were non-Hispanic white, 11.8% (0.5%) were non-Hispanic black, 15.9% (0.7%) were Hispanic, and 7.2% (0.3%) were Asian and other. An estimated 88.9% (0.3%) of participants had completed a high school education, 89.9% (0.3%) had health insurance, 6.6% (0.3%) reported a history of GDM, 26.8% (0.3%) had hypertension, and 65.3% (0.3%) were overweight or obese.

Among civilian, noninstitutionalized adults reporting no physician diagnosis of diabetes (Table 1), an estimated 80.0 million, or 36.0%, were at high risk of diabetes based on a physician diagnosis of prediabetes or an elevated ADA risk score. Of these, 17.9 million (22.2% of those at high risk or 8.0% overall) had diagnosed prediabetes, 73.3 million (91.9% of those at high risk or 33.3% overall) had an elevated ADA risk score, and 11.3 million (14.1% of those at high risk or 5.1% overall) had both. Among 145.5 million Americans with elevated BMI (overweight or obese) who would be eligible for diabetes prevention services, 68.1 million were considered at high risk based on reported prediabetes diagnoses (14.6 million) or an elevated ADA risk score (53.5 million).

**Table 1.** Number of US Adults With Diagnosed Prediabetes, Positive ADA Risk Test Findings, or Both^a^

ADA Risk Test Finding^b^	Diagnosed Prediabetes, 1 Million Population (95% CI)		Total, 1 Million Population (95% CI)
	Yes	No	
All adults			
Positive	11.3 (10.7-11.8)	62.1 (60.7-63.5)	73.3 (71.9-74.8)
Negative	6.6 (6.1-7.0)	143.0 (141.3-144.7)	149.6 (147.9-151.4)
Total	17.9 (16.2-19.5)	205.1 (204.1-206.2)	223.0 (222.2-223.8)
Adults with elevated BMI^c^			
Positive	10.2 (9.7-10.7)	53.5 (52.2-54.8)	63.7 (62.3-65.0)
Negative	4.5 (4.1-4.8)	77.3 (75.8-78.8)	81.8 (80.0-83.3)
Total	14.6 (14.0-15.3)	130.8 (129.4-132.2)	145.5 (144.0-146.9)

Abbreviations: ADA, American Diabetes Association; BMI, body mass index (calculated as weight in kilograms divided by height in meters squared).

^a^ Data are from the National Health Interview Survey, 2016 to 2017, and include US adults with and without elevated BMI and without diagnosed diabetes. Numbers were calculated using the July 1, 2017, US Census estimates for the civilian, noninstitutionalized population.

^b^ The ADA composite score^22^ of diabetes risk factors includes age, sex, race/ethnicity, family history of diabetes, BMI, history of gestational diabetes mellitus, diagnosed hypertension, and lack of physical activity. A score of 5 or higher (possible range, 0-11) was considered high risk for diabetes.

^c^ Elevated BMI is defined as 23.0 or higher for Asian adults and 25.0 or higher for all other adults.

Compared with US adults with elevated BMI who are not at high risk, adults with diagnosed prediabetes and those with ADA scores of at least 5 without a prediabetes diagnosis, were older (mean, 54.5 [0.3] and 60.1 [0.2] years, respectively, vs 36.8 [0.1] years), less likely to have attained education beyond high school (61.0% [1.0%] and 59.7% [0.7%], respectively, vs 66.8% [0.6%]), more likely to be insured (93.9% [0.6%] and 92.8% [0.8%], respectively, vs 86.6% [0.5%]), and more likely to report hypertension (56.4% [1.2%] and 56.8% [0.6%], respectively, vs 10.5% [0.3%]) (Table 2). Women with diagnosed prediabetes had a much higher reported prevalence of GDM (19.1% [1.5%]) compared with women with elevated ADA risk scores (7.1% [0.5%]) and those not at high risk (5.2% [0.4%]).

**Table 2.** Characteristics of US Adults With Elevated BMI and No Diagnosed Diabetes^a^

Characteristic	Total (n = 33 078)	Not at High Risk (n = 15 656)	Diagnosed Prediabetes (n = 3503)^b^	High ADA Risk Score (n = 13 919)^c^
Weighted population, 1 million (SE)^d^	145.5 (0.7)	77.3 (0.8)	14.6 (0.3)	53.5 (0.6)
Age group, y				
18-44	46.1 (0.5)	74.2 (0.5)	26.8 (1.0)	10.9 (0.4)
45-64	36.7 (0.4)	23.8 (0.5)	45.9 (1.1)	52.8 (0.6)
≥65	17.2 (0.3)	2.0 (0.1)	27.3 (0.9)	36.3 (0.6)
Age, mean (SE), y	47.2 (0.2)	36.8 (0.1)	54.5 (0.3)	60.1 (0.2)
Male	52.6 (0.4)	50.2 (0.5)	46.1 (1.2)	57.8 (0.5)
Race/ethnicity				
Non-Hispanic white	63.1 (0.9)	57.5 (1.0)	61.4 (1.4)	71.7 (0.9)
Non-Hispanic black	12.7 (0.5)	13.1 (0.6)	14.1 (0.9)	11.9 (0.6)
Non-Hispanic Asian	5.9 (0.3)	7.6 (0.4)	6.6 (0.7)	3.2 (0.3)
Hispanic	17.2 (0.8)	20.7 (0.9)	16.7 (1.2)	12.2 (0.8)
Other	1.1 (0.1)	1.1 (0.1)	1.3 (0.3)	1.0 (0.1)
Educational attainment^e^				
<High school	11.5 (0.3)	10.1 (0.4)	13.1 (0.8)	13.0 (0.5)
Completed high school^f^	24.9 (0.4)	23.1 (0.5)	25.9 (1.0)	27.3 (0.5)
>High school	63.6 (0.5)	66.8 (0.6)	61.0 (1.0)	59.7 (0.7)
Insurance^e^	89.6 (0.3)	86.6 (0.5)	93.9 (0.6)	92.8 (0.8)
History of GDM^g^	7.6 (0.3)	5.2 (0.4)	19.1 (1.5)	7.1 (0.5)
Self-reported hypertension status^e^	32.2 (0.4)	10.5 (0.3)	56.4 (1.2)	56.8 (0.6)
Weight status^h^				
Overweight	54.3 (0.4)	63.8 (0.5)	40.6 (1.1)	44.3 (0.6)
Obesity	45.7 (0.4)	36.2 (0.5)	59.4 (1.1)	55.7 (0.6)
BMI, mean (SE)	33.7 (0.1)	32.2 (0.1)	35.2 (0.3)	35.4 (0.2)

Abbreviations: ADA, American Diabetes Association; BMI, body mass index (calculated as weight in kilograms divided by height in meters squared); GDM, gestational diabetes; SE, standard error.

^a^ Data are from the National Health Interview Survey, 2016 to 2017. *P* < .01 for all pairwise comparisons conducted using χ^2^ or 2-tailed *t* test, except where indicated. Elevated BMI is defined as 23.0 or higher for Asian adults and 25.0 or higher for all other adults. Unless otherwise indicated, data are expressed as percentage (SE).

^b^ Defined by respondents’ self-reporting physician diagnosis of prediabetes.

^c^ Indicates ADA risk score of at least 5, without diagnosed prediabetes. Risk score is described in eTable 1 in the Supplement.

^d^ Population size calculated using the July 1, 2017, estimates of the civilian, noninstitutionalized population from the US Census Bureau.

^e^ *P* ≥ .05 for comparison between groups with diagnosed prediabetes and high ADA risk score.

^f^ Includes completion of General Educational Development.

^g^ Among women only.

^h^ Overweight defined as a BMI of 23.0 to 29.9 for Asian adults and 25.0 to 29.9 for all other adults; obesity, BMI of 30.0 or higher.

Adults with elevated BMI who were at high risk for diabetes (by ADA risk score or diagnosed prediabetes) were 1.5 to 2.0 times as likely as those with elevated BMI but not at high risk to undergo testing of glucose levels or to receive advice from health care professionals about activities and/or programs to lower diabetes risk (Table 3).

**Table 3.** Screening, Advice/Referrals, and Engagement in Diabetes Prevention Activities by US Adults With Elevated Body Mass Index and No Diagnosed Diabetes^a^

	Population^b^			
	Not High Risk (n = 15 656)	Diagnosed Prediabetes (n = 3503)^c^	High ADA Risk Score (n = 13 919)^d^
Weighted population size, 1 million (95% CI)^e^	77.3 (75.8-78.8)	14.6 (14.0-15.3)	53.5 (52.2-54.8)
Received blood test for high glucose levels			
<1 y ago	48.3 (47.1-49.5)	81.0 (79.1-82.7)	69.8 (68.6-70.9)
1-3 y ago	21.4 (20.5-22.3)	13.0 (11.5-14.8)	14.6 (13.8-15.4)
>3 y ago	11.0 (10.4-11.7)	4.9 (4.1-5.9)	6.6 (6.1-7.2)
Never	16.5 (15.6-17.5)	0.8 (0.5-1.3)	6.5 (5.9-7.2)
Do not know	2.7 (2.3-3.1)	0.3 (0.2-0.5)	2.5 (2.1-2.9)
Advice by health care professionals in the past year			
Increase physical activity	27.5 (26.6-28.5)	63.0 (60.9-65.1)	42.3 (41.2-43.4)
Reduce fat or calorie content in diet	22.7 (21.8-23.6)	59.2 (57.0-61.3)	35.6 (34.4-36.7)
Participate in weight loss program	6.6 (6.1-7.1)	21.3 (19.6-23.2)	10.7 (10.0-11.4)
Participate in program to prevent diabetes	0.3 (0.2-0.4)	4.9 (4.1-6.0)	0.4 (0.3-0.5)
Any of the above	33.1 (32.1-34.2)	73.5 (71.6-75.3)	50.6 (49.5-51.8)
Followed advice of health professionals^f^			
Increased physical activity	74.9 (73.2-76.6)	70.0 (67.4-72.5)	66.5 (64.9-68.1)
Reduced fat or calorie content in diet	76.7 (74.7-78.6)	75.8 (73.2-78.3)	75.2 (73.4-76.9)
Participated in weight loss program	36.9 (33.0-41.0)	35.0 (30.5-39.8)	33.5 (30.1-37.0)
Participated in program to prevent diabetes	30.5 (16.0-50.2)	39.6 (30.7-49.2)	40.4 (27.5-54.7)
Engaged in any behavior	86.1 (84.8-87.3)	85.8 (84.0-87.5)	81.8 (80.6-83.0)
Use of oral medication	NA	14.5 (13.1-16.0)	NA

Abbreviations: ADA, American Diabetes Association; NA, not applicable.

^a^ Elevated BMI (calculated as weight in kilograms divided by height in meters squared) indicates 23.0 or higher for Asian adults and 25.0 or higher for all other adults. Data are from the National Health Interview Survey, 2016 to 2017.

^b^ Unless otherwise indicated, data are expressed as percentage (95% CI).

^c^ Defined by respondents’ self-reporting physician diagnosis of prediabetes.

^d^ Indicates ADA risk score of at least 5, without diagnosed prediabetes. Risk score is described in eTable 1 in the Supplement.

^e^ Calculated using July 1, 2017, estimates of the civilian, noninstitutionalized population from the US Census Bureau.

^f^ Includes those who received advice in the past year.

Among 14.6 million adults with elevated BMI and diagnosed prediabetes (weighted numbers are shown in the Figure, percentages in Table 3, and numbers in eTable 4 in the Supplement), 81.0% (95% CI, 79.1%-82.7%) reported undergoing a glucose test in the past year, and an additional 13.0% (95% CI, 11.5%-14.8%) reported undergoing a glucose test in the past 1 to 3 years. An estimated 73.5% (95% CI, 71.6%-75.3%) reported receiving any guidance in the past year from their health care professional about activities and/or programs to lower diabetes risk. Specifically, 63.0% (95% CI, 60.9%-65.1%) reported receiving advice from a health care professional to increase physical activity; 59.2% (95% CI, 57.0%-61.3%), to reduce fat or caloric intake; and 21.3% (95% CI, 19.6%-23.2%), to participate in a weight loss program. In addition, 4.9% (95% CI, 4.1%-6.0%) reported receiving a referral to a diabetes prevention LSM program. Of those advised, 70.0% (95% CI, 67.4%-72.5%) reported increasing physical activity; 75.8% (95% CI, 73.2%-78.3%), reducing dietary fat or calorie intake; 35.0% (95% CI, 30.5%-39.8%), participating in weight loss programs; and 39.6% (95% CI, 30.7%-49.2%), participating in programs to prevent diabetes. An estimated 14.5% (95% CI, 13.1%-16.0%) reported taking oral medication to lower blood glucose levels.

**Figure.**
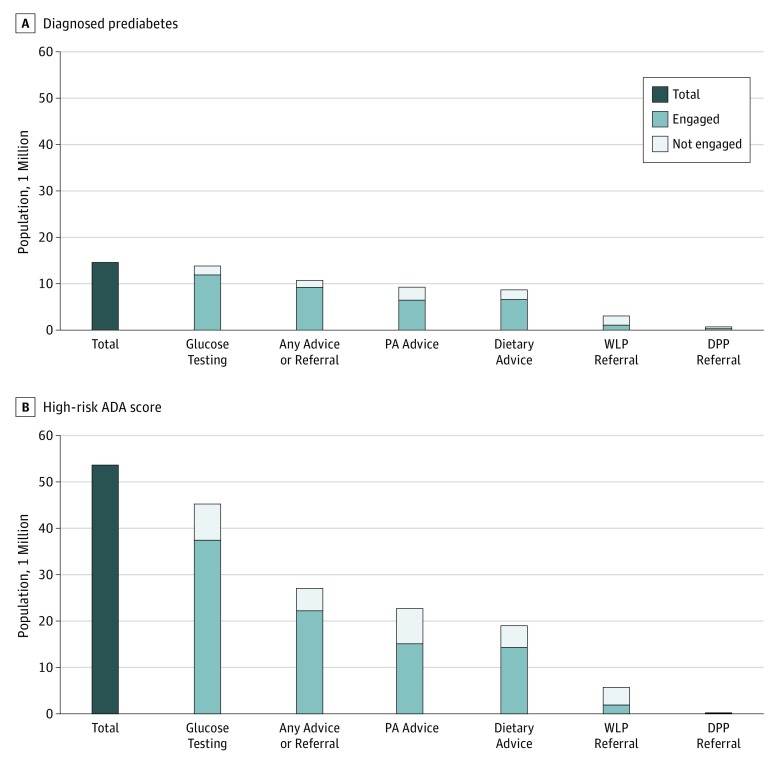
The Diabetes Prevention Continuum Graphs depict the numbers of US adults with elevated body mass index (calculated as weight in kilograms divided by height in meters squared). Total bars represent those eligible for lifestyle modification programs. Subsequeent bar heights depict eligible adults who reported receiving screening, referral, or advice regarding diabetes prevention behaviors; these bars were divided further to illustrate the number of those who did and did not engage among those advised or referred. Data are from the National Health Interview Survey, 2016 to 2017. Overweight was defined as a body mass index from 23.0 to 29.9 for Asian adults and 25.0 to 29.9 for all other adults; obesity, body mass index of 30.0 or higher. Output in eTable 4 in the Supplement was used for figure development. DPP indicates Diabetes Prevention Program; PA, physical activity; WLP, weight loss program.

In comparison, of 53.5 million adults with elevated BMI and high ADA risk scores but no prediabetes diagnosis (weighted numbers are shown in the Figure, percentages in Table 3, and numbers in eTable 4 in the Supplement), lower overall proportions (69.8% [95% CI, 68.6%-70.9%]) reported undergoing glucose testing in the past year; an additional 14.6% (95% CI, 13.8%-15.4%) reported undergoing testing in the past 1 to 3 years. Half of those with elevated BMI and ADA risk scores reported receiving any diabetes risk–reduction advice or referral (50.6%; 95% CI, 49.5%-51.8%). Specifically, 42.3% (95% CI, 41.2%-43.4%) were advised to be more physically active; 35.6% (95% CI, 34.4%-36.7%), to reduce fat or caloric intake; 10.7% (95% CI, 10.0%-11.4%), to participate in weight loss programs; and 0.4% (95% CI, 0.3%-0.5%), to join diabetes prevention programs. Among those advised, in the past year, 66.5% (95% CI, 64.9%-68.1%) reported increasing physical activity; 75.2% (95% CI, 73.4%-76.9%), reducing dietary fat and calories consumed; 33.5% (95% CI, 30.1%-37.0%), participating in weight loss programs; and 40.4% (95% CI, 27.5%-54.7%), participating in programs to prevent diabetes.

In relative terms, engagement in diabetes risk–reducing activities and/or programs was higher among those receiving advice and/or referral from a health care professional (those with diagnosed prediabetes, 85.8% [95% CI, 84.0%-87.5%]; those with elevated risk scores, 81.8% [95% CI, 80.6%-83.0%]) compared with those not receiving such advice and/or referrals (those with diagnosed prediabetes, 62.3% [95% CI, 58.6%-65.9%]; those with elevated ADA risk scores, 50.5% [95% CI, 48.6%-52.5%]) (Table 4). Patterns of engagement in any diabetes risk–reduction activities and/or programs across sociodemographic or clinical characteristics by people with elevated BMI and at high risk for diabetes were mixed. Greater engagement across groups was generally seen for adults aged 45 to 64 years, non-Hispanic black and Asian adults, those with hypertension, those with a history of GDM in pregnancy, and those with more than a high school–level education, compared with their respective counterparts. Notably, among those without diagnosed prediabetes and not advised or referred regarding LSM, a higher likelihood of engagement was seen in 2017 than in 2016 (52.8% [95% CI, 50.2%-55.3%] vs 48.6% [95% CI, 46.2%-51.0%]).

**Table 4.** Engagement in Any Diabetes Risk-Reducing Activity in Past Year Among US Adults Aged 18 Years or Older With Elevated BMI and at High Risk for Diabetes^a^

Sociodemographic or Clinical Subgroup	Weighted Population Engaged, % (95% CI)					
	Diagnosed Prediabetes^b^		High ADA Risk Score^c^		
	Received No Advice and/or Referral	Received Advice and/or Referral	Received No Advice and/or Referral	Received Advice and/or Referral
Overall	62.3 (58.6-65.9)	85.8 (84.0-87.5)	50.5 (48.6-52.5)	81.8 (80.6-83.0)
Year				
2016	63.8 (58.8-68.5)	86.5 (83.9-88.8)	48.6 (46.2-51.0)	80.8 (79.1-82.4)
2017	60.7 (55.2-66.0)	85.2 (82.6-87.5)	52.8 (50.2-55.3)	82.7 (81.0-84.4)
*P* value	.40	.45	.007	.11
Age group, y				
18-44	68.6 (60.5-75.8)	82.1 (77.2-86.1)	44.4 (39.0-49.9)	78.6 (73.7-82.8)
45-64	59.3 (53.0-65.3)	88.9 (86.5-91.0)	53.1 (50.6-55.6)	83.2 (81.5-84.7)
≥65	60.8 (54.5-66.7)	84.0 (80.5-86.9)	48.9 (46.4-51.4)	80.5 (78.5-82.4)
*P* value	.18	.003	.001	.04
Sex				
Male	61.0 (55.3-66.4)	85.1 (82.0-87.7)	49.4 (47.1-51.8)	80.7 (78.9-82.5)
Female	63.6 (58.4-68.6)	86.5 (83.9-88.7)	52.4 (49.6-55.2)	83.0 (81.3-84.6)
*P* value	.50	.47	.08	.07
Race/ethnicity^d^				
Non-Hispanic white	63.9 (59.4-68.1)	84.4 (82.1-86.5)	51.0 (48.8-53.2)	80.1 (78.7-81.5)
Non-Hispanic black	60.6 (49.2-71.0)	89.5 (83.4-93.5)	50.2 (45.2-55.2)	84.7 (80.9-87.8)
Non-Hispanic Asian	51.3 (32.2-70.0)	93.8 (87.4-97.1)	40.2 (31.3-49.8)	90.9 (85.4-94.5)
Hispanic	61.0 (49.5-71.4)	84.0 (78.0-88.6)	50.0 (44.2-55.9)	85.5 (81.7-88.6)
*P* value	.74	.04	.21	<.001
Educational attainment				
<High school	48.7 (37.3-60.3)	85.7 (80.4-89.7)	35.7 (31.7-40.0)	74.6 (70.0-78.6)
Completed high school^e^	52.1 (44.3-59.8)	82.7 (78.4-86.4)	44.6 (41.7-47.6)	78.5 (76.0-80.9)
>High school	69.8 (65.2-74.1)	87.2 (85.0-89.1)	57.4 (55.1-59.7)	84.4 (83.0-85.7)
*P* value	<.001	.11	<.001	<.001
Insurance				
No	63.0 (49.2-75.0)	83.8 (72.9-90.9)	45.7 (40.4-51.0)	71.5 (63.2-78.6)
Yes	62.3 (58.3-66.1)	86.0 (84.0-87.7)	51.0 (49.0-53.1)	82.3 (81.1-83.5)
*P* value	.92	.63	.05	.002
History of GDM				
No	62.7 (58.8-66.5)	85.4 (83.4-87.2)	50.2 (48.2-52.1)	81.8 (80.6-83.0)
Yes	59.2 (44.2-72.7)	91.1 (83.8-95.3)	64.4 (54.0-73.6)	81.9 (74.0-87.8)
*P* value	.65	.12	.008	.98
Self-reported hypertension status				
No	60.2 (54.8-65.4)	84.0 (80.8-86.7)	48.0 (45.5-50.5)	81.0 (78.8-83.0)
Yes	64.5 (58.9-69.7)	87.1 (84.6-89.2)	53.2 (50.7-55.6)	82.3 (80.8-83.7)
*P* value	.29	.11	.001	.32
BMI^f^				
Overweight	62.2 (56.9-67.1)	85.5 (82-88.5)	48.7 (46.2-51.1)	81.7 (79.7-83.5)
Obese	62.5 (56.4-68.3)	86.0 (83.8-87.9)	52.6 (50-55.2)	81.9 (80.3-83.3)
*P* value	.93	.82	.02	.87

Abbreviations: ADA, American Diabetes Association; BMI, body mass index (calculated as weight in kilograms divided by height in meters squared); GDM, gestational diabetes mellitus.

^a^ Risk-reducing activity defined as self-reporting engaging in dietary changes or physical activity changes in the past year to prevent diabetes or attending a weight loss program or a diabetes prevention program. Received Advice and/or Referral indicates participant reported that his or her health professional guided him or her to engage in a diabetes risk–reducing activity or program. Elevated BMI was defined as 23.0 or more for Asian adults and 25.0 or more for all other adults. All estimates are weighted percentages and 95% CIs in parentheses calculated from multivariable logistic regression, controlling for all other variables. *P* values were calculated from an adjusted Wald *F* test for the associations between independent variables and engagement in any diabetes risk-reducing activity.

^b^ Defined by respondents’ self-reporting physician diagnosis of prediabetes.

^c^ Indicates ADA risk score of at least 5, without diagnosed prediabetes. Risk score is described in eTable 1 in the Supplement.

^d^ Estimates for adults of other races/ethnicities are not shown.

^e^ Includes completion of General Educational Development.

^f^ Overweight was defined as a BMI of 23.0 to 29.9 for Asian adults and 25.0 to 29.9 for all other adults; obesity, BMI of 30.0 or higher.

In sensitivity analyses that included all adults without diabetes (eTable 5 in the Supplement)—17.9 million with prediabetes and 62.1 million without prediabetes but with elevated ADA risk scores—patterns and estimates regarding testing of glucose levels, advice and/or referrals by health care professionals, and engagement in diabetes risk–reduction activities and/or programs were similar to those of analyses restricted to adults who were overweight or had obesity. An estimated 85% to 90% of those at high risk reported undergoing a blood glucose test in the past 3 years; 68.6% of those with diagnosed prediabetes (95% CI, 66.8%-70.4%; range, 5.0%-58.3%) and 47.6% of those with elevated ADA risk scores (95% CI, 46.5%-48.7%; range, 0.4%-39.8%) reported receiving any advice or referral regarding diabetes prevention activities/programs. Of these, 35.7% to 75.9% of adults with diagnosed prediabetes and 33.2% to 75.4% of those with elevated ADA scores reported engaging in these activities.

In sensitivity analyses to estimate proportions of respondents who engaged in each specific activity or program separately and variation across sociodemographic and clinical characteristics (eTable 6 in the Supplement), we noted greater engagement among those with greater educational attainment, those who were middle aged, and those with insurance, a history of GDM, hypertension, or obesity. In addition, greater engagement appeared to occur among women and minority races/ethnicities.

## Discussion

These data provide, to our knowledge, the most comprehensive assessment of the degree to which US adults who are likely to benefit from diabetes prevention services are undergoing testing, counseling, and actual engagement in risk-reduction activities and/or programs. One-fifth of those with an elevated ADA risk score in 2017 reported a formal prediabetes diagnosis. Individuals with prediabetes diagnoses were more likely than those without to receive diabetes risk–reduction advice and/or referrals by health care professionals. Advice and/or referral by a health care professional was associated with a higher likelihood of participation. Overall, engagement in LSM programs designated for diabetes prevention was exceedingly low among all high-risk US adults.

The backdrop of health reforms^26^ and large-scale efforts and investments directed toward diabetes prevention in the United States,^27^ the United Kingdom,^28^ and other countries accentuate the magnitude and importance of this issue. Our findings suggest that efforts to expand the supply of diabetes prevention LSM programs are, at least now, insufficient, not being matched by uptake, or both. Our population prevention continuum identified 3 specific gaps that are potentially modifiable.

First, 1 in 5 adults at high risk for diabetes reported a prediabetes diagnosis. Of the remaining 53.5 million who were overweight or obese with elevated ADA risk scores, 85% to 90% underwent a blood glucose test in the past 3 years. Therefore, people at high risk may not meet biochemical prediabetes thresholds,^23^ or factors involving the test (eg, inaccuracy of laboratory findings), health care professional (eg, poor recognition, poor communication, or inaction), respondent (eg, recall bias), or some combination of these may be at play. This is important because our data suggest that prediabetes diagnoses may lead to a higher likelihood of counseling or referral by health care professionals.

Second, health care professionals were 2 to 3 times more likely to give general physical activity or dietary advice to patients than refer them to formal programs. Even among respondents with elevated BMI and diagnosed prediabetes, general advice about diabetes risk reduction (approximately 60%) was far more common than referral to a weight loss (approximately 20%) or a diabetes prevention (approximately 5%) program. Health care professionals may not believe in the effectiveness and cost-benefit of diabetes prevention programs, may be less aware of these programs, or may believe their patients would have less accessibility to programs.^29,30^ Indeed, prediabetes constitutes a large heterogeneous group with varied risk levels. Because the motivating results from diabetes prevention trials were mostly observed in individuals with impaired glucose tolerance, health care professionals may be less inclined to refer people with lower-risk prediabetes subtypes. Furthermore, the proportions were even lower for adults without a prediabetes diagnosis, which may mean that health care professionals are more concerned about the long-term costs^31,32^ and comorbidities^33^ among people with biochemical evidence of prediabetes than among those without.

Third, among those advised or referred, sizeable gaps occurred between the 66% to 76% (or 40%-60% overall) of US adults at high risk of diabetes reporting some LSM in the past year compared with the 33% to 40% of those advised (or less than 10% overall) attending a formal program. Our study cannot assertively explain low levels of program engagement. Insurance coverage may influence engagement in diabetes risk–reducing activities, but insufficient power was available to examine whether coverage was associated with program engagement specifically. Competing priorities may also be barriers to engagement,^34^ especially among young adults and especially if participants do not perceive a benefit that outweighs the costs and time to participate in diabetes prevention programs. Comorbidities and higher self-perceived risk—notably, in the case of those with a history of GDM, hypertension, or obesity—appeared to motivate participation in risk-reducing activities, especially among those not advised or referred.

Given that barriers associated with these gaps are likely multifactorial, a variety of concurrent implementation processes and supportive policies may increase the supply and coverage of programs, awareness (among those at risk and health care professionals), initial engagement, and retention. Investments in each of these areas (eg, Ad Council campaign,^35^ the American Medical Association’s efforts to increase awareness among health care professionals,^36^ and others) may increase awareness. Further research may also be beneficial, especially in terms of increasing engagement and effectiveness of programs, particularly through leveraging behavioral economics.^37,38^

To assist with initial referral and engagement in busy and increasingly complex clinical care, decision-support technologies may be useful reminder prompts to physicians to test, counsel, and refer patients to lifestyle change programs. As an example,^39^ in New York City, New York, integration of decision-support tools into electronic health records to prompt health care professionals to screen for prediabetes and diabetes was associated with the doubling of test rates among eligible individuals (from approximately 10% to >20%); notably, this increase was also associated with higher test rates among ineligible individuals. Another example of within-clinic prompting^40^ is the integration of Exercise as a Vital Sign into clinical workflows. Compared with clinics without Exercise as a Vital Sign, documentation of physical activity was nominally higher (26.2% vs 23.7% of visits), but referral of at-risk patients to LSM programs (2.1% vs 1.7%) was only slightly higher for participating clinics and still very low overall. So far, on average, decision-support tools appear to offer modest benefits, but for certain segments of health care professionals, the associations may be larger.

Evidence from natural experiment studies show small associations between employer and health plan policies to facilitate diabetes prevention and engagement of adults at high risk for diabetes.^41^ With regard to financial incentives, studies show associations between incentives directly given to respondents and better initial weight loss, but no improvement in maintenance of weight loss.^42,43^ Studying what psychological, economic, and time preferences motivate people at high risk to engage remains an important area of study^37,38^ and of high value to payers and organizations delivering LSM programs.

Despite data regarding the efficacy of metformin in reducing diabetes incidence,^3^ 14.5% of those with prediabetes reported using medications. Low use of metformin^44^ and other medications to prevent diabetes may be associated with patient or health care professional preferences, concerns about adverse effects, and possibly consideration that medications to prevent diabetes likely will have to be taken in perpetuity because the effect wears off after discontinuation.^6^

Previous national reports showing low achievement of diabetes care goals^45,46^ were followed by subsequent improvements over time.^33,47^ In addition, those with diagnosed diabetes tend to experience more aggressive treatment and achievement of care goals.^33^ These findings highlight the value of this report as a benchmark from which to monitor future program availability and coverage, identification of prediabetes, referral, and retention as these programs mature. Furthermore, quality measures proposed by the American Medical Association’s Prediabetes Quality Measures Technical Expert Panel^48^ may prompt even greater accountability, action, and improvement.

### Limitations

We used cross-sectional data that represented respondents’ activities and recall at the time of the survey. If someone was diagnosed with prediabetes several years ago and made lifestyle changes then, these would not get enumerated in the surveys. Self-reported data, especially for height and weight, can be subject to recall or social desirability biases, which may have influenced estimates of those with elevated ADA risk scores. The ADA risk score itself does not have perfect associations with who will develop diabetes. Our definitions of high risk for diabetes may be overly sensitive, although our estimates of the total numbers of adults at high risk align with national data based on biochemical testing to confirm high risk for diabetes.^2,49^ Respondents may have misinterpreted what was meant regarding diabetes programs in the questions posed. Our estimates of engagement in LSM programs for diabetes prevention do not reflect whether respondents attended a single session or completed the program because they were only asked about participation.

## Conclusions

Our analyses used 2 recent waves of nationally representative surveys with large sample sizes offering a contemporary perspective on diabetes prevention in the United States. Our study offers a first assessment of participant-reported advice and/or referral by their health care professionals and engagement by adults at high risk using a national diabetes prevention continuum, demonstrating where the gaps occur, providing insights into possible policy and program actions,^26,50^ and providing a benchmark for future population-level monitoring.

## Notes

Supplement. **eTable 1.** Risk Factors for Type 2 Diabetes as Assessed in the National Health Interview Surveys, 2016 and 2017 **eTable 2.** Questions Regarding Diabetes Prevention Services in the National Health Interview Survey 2016 **eTable 3.** Characteristics of US Adults Aged ≥18 Years Without Diagnosed Diabetes, National Health Interview Survey 2016-2017 **eTable 4.** Numbers of US Adults (in Millions) With Elevated Body Mass Index Eligible for Lifestyle Modification Programs Who Received Screening, Referral, or Advice Regarding Diabetes Prevention Behaviors and Numbers Engaging, National Health Interview Survey 2016-2017 **eTable 5.** Receipt of Screening and Advice and Engagement in Diabetes Prevention Activities by US Adults Aged ≥18 Years Without Diagnosed Diabetes, National Health Interview Survey 2016-2017 **eTable 6.** Engagement in Specific Diabetes Prevention Activities by US Adults Aged ≥18 Years With Elevated Body Mass Index and High Risk for Diabetes but No Diagnosed Diabetes, National Health Interview Survey 2016-2017
