# Supplementary material for: Reach and Use of Diabetes Prevention Services in the United States, 2016-2017
Source: JAMA Netw Open. 2019 May 10;2(5):e193160. doi: 10.1001/jamanetworkopen.2019.3160 (PMC6512285; doi:10.1001/jamanetworkopen.2019.3160)
Supplement: Supplement. — eTable 1. Risk Factors for Type 2 Diabetes as Assessed in the National Health Interview Surveys, 2016 and 2017 eTable 2. Questions Regarding Diabetes Prevention Services in the National Health Interview Survey 2016 eTable 3. Characteristics of US Adults Aged ≥18 Years Without Diagnosed Diabetes, National Health Interview Survey 2016-2017 eTable 4. Numbers of US Adults (in Millions) With Elevated Body Mass Index Eligible for Lifestyle Modification Programs Who Received Screening, Referral, or Advice Regarding Diabetes Prevention Behaviors and Numbers Engaging, National Health Interview Survey 2016-2017 eTable 5. Receipt of Screening and Advice and Engagement in Diabetes Prevention Activities by US Adults Aged ≥18 Years Without Diagnosed Diabetes, National Health Interview Survey 2016-2017 eTable 6. Engagement in Specific Diabetes Prevention Activities by US Adults Aged ≥18 Years With Elevated Body Mass Index and High Risk for Diabetes but No Diagnosed Diabetes, National Health Interview Survey 2016-2017 [file jamanetwopen-2-e193160-s001.pdf]

Supplementary Online Content

Ali MK, Bullard KM, Imperatore G, et al. Reach and use of diabetes prevention services in the United States, 2016-2017. *JAMA Netw Open*. 2019;2(5):e193160. doi:10.1001/jamanetworkopen.2019.3160

eTable 1. Risk Factors for Type 2 Diabetes as Assessed in the National Health Interview Surveys, 2016 and 2017

eTable 2. Questions Regarding Diabetes Prevention Services in the National Health Interview Survey 2016

eTable 3. Characteristics of US Adults Aged ≥18 Years Without Diagnosed Diabetes, National Health Interview Survey 2016-2017

eTable 4. Numbers of US Adults (in Millions) With Elevated Body Mass Index Eligible for Lifestyle Modification Programs Who Received Screening, Referral, or Advice Regarding Diabetes Prevention Behaviors and Numbers Engaging, National Health Interview Survey 2016-2017

eTable 5. Receipt of Screening and Advice and Engagement in Diabetes Prevention Activities by US Adults Aged ≥18 Years Without Diagnosed Diabetes, National Health Interview Survey 2016-2017

eTable 6. Engagement in Specific Diabetes Prevention Activities by US Adults Aged ≥18 Years With Elevated Body Mass Index and High Risk for Diabetes but No Diagnosed Diabetes, National Health Interview Survey 2016-2017

This supplementary material has been provided by the authors to give readers additional information about their work.

© 2019 Ali MK et al. *JAMA Network Open.*

eTable 1. Risk factors for Type 2 Diabetes as Assessed in the National Health Interview Surveys, 2016 and 2017

Risk factors for diabetes	NHIS Questionnaire items	Component score
Age	What is your age?	0: 18-39y<br>1: 40-49y<br>2: 50-59y<br>3: ≥60y
Sex	Are you male or female?	0: Female<br>1: Male
Family history of diabetes	Has your mother, father, brother, or sister EVER been told by a doctor or other health professional that they have diabetes or sugar diabetes?	0: No<br>1: Yes
History of gestational diabetes	Were you EVER told by a doctor or other health professional that you had diabetes, sugar diabetes, or gestational diabetes during pregnancy?	0: No<br>1: Yes
Diagnosed hypertension	Have you EVER been told by a doctor or other health professional that you had hypertension, also called high blood pressure?	0: No<br>1: Yes
Lack of physical activity	How often do you do VIGOROUS leisure-time physical activities for AT LEAST 10 MINUTES that cause HEAVY sweating or LARGE increases in breathing or heart rate?<br><br>How often do you do LIGHT OR MODERATE LEISURE-TIME physical activities for AT LEAST 10 MINUTES that cause ONLY LIGHT sweating or a SLIGHT to MODERATE increase in breathing or heart rate?	0: No<br>1: Never (both items)
Self-reported height and weight	How much do you weigh without shoes?<br><br>How tall are you without shoes?	0—3, according to combination of self-reported height and weight

Accessed at https://www.cdc.gov/nchs/nhis/data-questionnaires-documentation.htm
Accessed at https://doihaveprediabetes.org/wp-content/uploads/2018/09/Prediabetes_PrintableRiskTestEnglish.pdf; Adapted from Bang et al., Ann Intern Med 151:775-783, 2009.
*race/ethnicity not included in ADA prediabetes risk test

© 2019 Ali MK et al. *JAMA Network Open.*

eTable 2. Questions Regarding Diabetes Prevention Services in the National Health Interview Survey 2016

Question ID	Variable Name	Question Text	Options
		During the past 12 months, have you been told by a doctor or health professional to do any of the following….	
ACN.155_00.010	DBHVPAY	Increase your physical activity or exercise?	1 Yes<br>2 No<br>7 Refused<br>9 Don't know
ACN.155_00.020	DBHVCLY	Reduce the amount of fat or calories in your diet?	1 Yes<br>2 No<br>7 Refused<br>9 Don't know
ACN.155_00.030	DBHVWLY	Participate in a weight loss program?	1 Yes<br>2 No<br>7 Refused<br>9 Don't know
		Are you now doing any of the following…	
ACN.155_00.040	DBHVPAN	Increasing your physical activity or exercise?	1 Yes<br>2 No<br>7 Refused<br>9 Don't know
ACN.155_00.050	DBHVCLN	Reducing the amount of fat or calories in your diet?	1 Yes<br>2 No<br>7 Refused<br>9 Don't know
ACN.155_00.060	DBHVWNL	Participating in a weight loss program?	1 Yes<br>2 No<br>7 Refused<br>9 Don't know
ACN.155_00.070	DIBREL	Has your mother, father, brother, or sister EVER been told by a doctor or other health professional that they have diabetes or sugar diabetes? (only include blood relatives; not step relatives or those unrelated by blood)	1 Yes<br>2 No<br>7 Refused<br>9 Don't know
ACN.160_00.000	DIBEV1	Other than during pregnancy, have you EVER been told by a doctor or other health professional that you have diabetes or sugar diabetes?	1 Yes<br>2 No<br>3 Borderline or prediabetes<br>7 Refused<br>9 Don't know
ACN.165_00.000	DIBPRE1	Have you EVER been told by a doctor or other health professional that you have any of the following: prediabetes, impaired	1 Yes<br>2 No<br>7 Refused<br>9 Don't know

© 2019 Ali MK et al. *JAMA Network Open.*

		fasting glucose, impaired glucose tolerance, borderline diabetes, or high blood sugar?	
ACN.167_00.010	DIBTEST	About how long has it been since you last had a blood test for high blood sugar or diabetes?	1 One year ago or less<br>2 More than one year, but not more than two years ago<br>3 More than two years, but not more than three years ago<br>4 More than three years ago<br>5 Never<br>7 Refused<br>9 Don't know
ACN.170_00.000	DIBAGE	How old were you when a doctor or health professional FIRST told you that you had diabetes or sugar diabetes?	000 thu 100 Age at which diagnosed<br>997 Refused<br>999 Don't know
ACN.175_00.010	DIBTYPE	What type of diabetes do you have?	1 Type 1<br>2 Type 2<br>3 Other<br>7 Refused<br>9 Don't know
ACN.180_00.000	DIBPILL	Are you NOW taking diabetic pills to lower your blood sugar? These are sometimes called oral agents or oral hypoglycemic agents.	1 Yes<br>2 No<br>7 Refused<br>9 Don't know
ACN.190_00.000	INSLN1	Insulin can be taken by shot or pump. Are you NOW taking insulin?	1 Yes<br>2 No<br>7 Refused<br>9 Don't know
ACN.190_00.010	DIBINS2	Thinking back to when you were first diagnosed with diabetes, how long was it before you started taking insulin?	1 Less than one month<br>2 One month to less than six months<br>3 Six months to less than one year<br>4 One year or more

© 2019 Ali MK et al. *JAMA Network Open.*

			7 Refused<br>9 Don’t know
ACN.190_00.020	DIBINS3	Since you started taking insulin, have you ever stopped taking it for more than 6 months?	1 Yes<br>2 No<br>7 Refused<br>9 Don’t know
ACN.190_00.030	DIBINS4	Was this only during the first year after you were diagnosed with diabetes?	1 Yes<br>2 No<br>7 Refused<br>9 Don’t know
ACN.195_00.010	DIBGDM	Were you FIRST (or EVER) told by a doctor or health profession that you had diabetes, sugar diabetes, or gestational diabetes during pregnancy?	1 Yes<br>2 No<br>7 Refused<br>9 Don’t know
ACN.197_00.010	DIBBABY	Have you EVER had a baby that weighed 9 pounds (4kg) or more at birth?	1 Yes<br>2 No<br>7 Refused<br>9 Don’t know
		These next questions are about a year-long program that can help prevent Type 2 diabetes. This program has weekly sessions during the first 6 months and month sessions over the last 6 months. People in the program receive support from a lifestyle coach on achieving and maintaining a healthy lifestyle.	
ACN.198_00.010	DIBPRGM	Have you EVER participated in this type of year-long program to prevent Type 2 diabetes?	1 Yes<br>2 No<br>7 Refused<br>9 Don’t know
ACN.198_00.020	DIBREFER	Has a doctor or other health care professional ever referred you to such a program to prevent Type 2 diabetes?	1 Yes<br>2 No<br>7 Refused<br>9 Don’t know
ACN.198_00.030	DIBBEGIN	How interested are you in beginning such a year -long program to prevent Type 2 diabetes? Would you say…	1 Very interested<br>2 Somewhat interested<br>3 Not interested<br>7 Refused<br>9 Don’t know

© 2019 Ali MK et al. *JAMA Network Open.*

eTable 3. Characteristics of US Adults Aged ≥18 Years Without Diagnosed Diabetes, National Health Interview Survey 2016-2017

	**Total**	**Not High Risk**	**Diagnosed Prediabetes***	**High ADA Risk score, No Diagnosed Prediabetes†**
**Sample size**	50912	30153	4272	16487
Weighted population, millions ‡	223.0 (0.4)	143.0 (0.9)	17.9 (0.9)	62.1 (0.7)
**Age group** §				
18-44 years, %	49.2 (0.4)	69.4 (0.4)	26.9 (1.0)	9.4 (0.3)
45-64 years, %	33.2 (0.3)	24.7 (0.4)	44.3 (1.0)	49.7 (0.5)
65+ years, %	17.5 (0.3)	5.9 (0.2)	28.8 (0.9)	40.9 (0.5)
**Age, years, mean (SE)**	46.1 (0.2)	38.2 (0.1)	54.7 (0.3)	61.6 (0.2)
**Male, %**	48.1 (0.3)	44.2 (0.4)	44.2 (1.0)	58.2 (0.5)
**Race/ethnicity** §				
NH white, %	65.1 (0.8)	62.4 (0.9)	62.7 (1.3)	72.1 (0.9)
NH black, %	11.8 (0.5)	11.6 (0.5)	13.1 (0.8)	12.0 (0.6)
NH Asian, %	6.2 (0.3)	7.3 (0.3)	6.7 (0.6)	3.4 (0.3)
Hispanic, %	15.9 (0.7)	17.7 (0.8)	16.2 (1.1)	11.6 (0.7)
Other, %	1.0 (0.1)	1.0 (0.1)	1.3 (0.3)	0.9 (0.1)
**Education**				
<HS, %	11.1 (0.3)	9.8 (0.3)	12.4 (0.7)	13.7 (0.5)
HS, %	24.1 (0.3)	22.3 (0.4)	25.5 (0.9)	27.8 (0.5)
>HS, %	64.8 (0.5)	67.9 (0.5)	62.1 (1.0)	58.4 (0.6)
**Insured, %** ‖	89.9 (0.3)	87.9 (0.4)	94.0 (0.5)	93.3 (0.3)
**History of GDM, %** ¶	6.6 (0.3)	4.6 (0.3)	18.9 (1.2)	6.9 (0.4)
**Hypertension, %**	26.8 (0.3)	9.5 (0.2)	53.7 (1.0)	58.5 (0.6)
**Weight status**				
Normal, %	34.7 (0.3)	45.9 (0.4)	17.9 (0.8)	13.8 (0.3)
Overweight, % **	35.4 (0.3)	34.5 (0.4)	33.3 (1.0)	38.2 (0.5)
Obese, %	29.9 (0.3)	19.6 (0.3)	48.8 (1.1)	48.0 (0.5)

© 2019 Ali MK et al. *JAMA Network Open.*

**BMI, (kg/m²) mean (SE)**	29.7 (0.1)	27.5 (0.1)	32.9 (0.3)	33.6 (0.2)

%, percent; ADA, American Diabetes Association; BMI, body mass index; GDM, gestational diabetes; HS, high school (including completion of General Educational Development), NH, non-Hispanic.

All estimates include standard errors in parentheses.

* Diagnosed prediabetes defined by respondents' self-reporting physician diagnosis of prediabetes.

† High-risk without a diagnosis of prediabetes defined by ADA score greater than or equal to 5.

‡ Population size calculated using the July 1, 2017 US resident civilian, noninstitutionalized population estimates from the US Census Bureau.

§ P-values ≥0.05 for comparison between diagnosed prediabetes and non-high risk

|| P-values ≥0.05 for comparison between diagnosed prediabetes and high ADA risk score (no diagnosed prediabetes)

¶ Among women only

** Overweight defined as body mass index 23.0–29.9 kg/m^2 for Asian adults and 25.0–29.9 kg/m^2 for all other adults; obesity defined as body mass index ≥30.0 kg/m^2.

© 2019 Ali MK et al. *JAMA Network Open.*

eTable 4. Numbers of US Adults (in Millions) With Elevated Body Mass Index* Eligible for Lifestyle Modification Programs Who Received Screening, Referral, or Advice Regarding Diabetes Prevention Behaviors and Numbers Engaging, National Health Interview Survey 2016-2017

		Engaged	Not engaged
Diagnosed Prediabetes	Total	14.6	
	Receive glucose test in past 3 years	11.9	1.9
	Any advice/referral	9.2	1.5
	PA advice	6.5	2.8
	Diet advice	6.6	2.1
	Weight loss program advice	1.1	2.0
	T2DM prevention program referral	0.3	0.4
ADA High Risk, no Diagnosed Prediabetes	Total	53.5	
	Receive glucose test in past 3 years	37.4	7.8
	Any advice/referral	22.2	4.9
	PA advice	15.1	7.6
	Diet advice	14.3	4.7
	Weight loss program advice	1.9	3.8
	T2DM prevention program referral	0.1	0.1

LSM, lifestyle modification; PA, physical activity; T2DM, type 2 diabetes mellitus.

Total of engaged and not engaged individuals equal to the number of people advised about diabetes risk reducing activities or referred to weight loss or diabetes prevention programs.

* Defined as body mass index ≥23 kg/m^2 for Asian adults and ≥25 kg/m^2 for all other adults

Data are shown in Figure 1

© 2019 Ali MK et al. *JAMA Network Open.*

eTable 5. Receipt of Screening and Advice and Engagement in Diabetes Prevention Activities by US Adults Aged ≥18 Years Without Diagnosed Diabetes, National Health Interview Survey 2016-2017

	Not High Risk	Diagnosed Prediabetes*	High ADA Risk Score, No Diagnosed Prediabetes †
**Sample size**	30153	4272	16487
Weighted population size, millions ‡	143.0 (0.9)	17.9 (0.9)	62.1 (0.7)
**Received blood test for high blood sugar**			
<1 year ago	47.9 (46.8, 48.9)	79.8 (78.1, 81.4)	69.9 (68.8, 71)
1-3 years ago	19.9 (19.2, 20.7)	13 (11.6, 14.5)	14.3 (13.6, 15.1)
>3 years ago	10.9 (10.4, 11.5)	5.8 (5, 6.7)	6.6 (6.1, 7.1)
Never	18.1 (17.3, 19)	1.0 (0.7, 1.5)	6.5 (5.9, 7.1)
Don't know	3.1 (2.8, 3.5)	0.3 (0.2, 0.6)	2.7 (2.3, 3.1)
**% Advised by health professionals in the past year to:**			
Increase physical activity	20.6 (20.0, 21.3)	58.3 (56.4, 60.1)	39.8 (38.8, 40.8)
Reduce fat or calorie content in diet	14.8 (14.2, 15.3)	52.6 (50.6, 54.6)	32.3 (31.3, 33.4)
Participate in weight loss program	3.8 (3.5, 4.2)	18.2 (16.7, 19.8)	9.4 (8.8, 10.0)
Participate in program to prevent type 2 diabetes	0.2 (0.2, 0.3)	5.0 (4.2, 6.0)	0.4 (0.3, 0.5)
Do any of the above	24.6 (23.9, 25.3)	68.6 (66.8, 70.4)	47.6 (46.5, 48.7)
**Among those advised by health professionals, % that:**			
Increased physical activity	72.8 (71.3, 74.3)	69.6 (67.1, 72.0)	66.3 (64.7, 67.8)
Reduced fat or calorie content in diet	76.5 (74.6, 78.2)	75.9 (73.4, 78.3)	75.4 (73.6, 77.1)
Participated in weight loss program	38.0 (34.2, 41.9)	35.7 (31.2, 40.4)	33.2 (29.9, 36.7)
Participated in program to prevent type 2 diabetes	29.8 (15.3, 49.9)	39.8 (31.5, 48.7)	41.7 (29.6, 55.0)
Engaged in any behavior among those receiving any advice or referral	78.5 (77.2, 79.7)	80.8 (78.7, 82.5)	76.0 (74.7, 77.2)
**Taking Oral Medication (%)**	N/A	13.8 (12.5, 15.1)	N/A

© 2019 Ali MK et al. *JAMA Network Open.*

ADA, American Diabetes Association; N/A, not applicable

All estimates are unadjusted weighted percentages with 95% confidence intervals in parentheses.

* Diagnosed prediabetes was defined by respondents' self-reporting physician diagnosis of prediabetes.

† High-risk without a diagnosis of prediabetes defined by ADA score greater than or equal to 5.

‡ Population size was calculated using July 1, 2017 US resident civilian, noninstitutionalized population estimates from US Census Bureau.

© 2019 Ali MK et al. *JAMA Network Open.*

eTable 6. Engagement in Specific Diabetes Prevention Activities by US Adults Aged ≥18 Years With Elevated Body Mass Index* and High Risk for Diabetes but no Diagnosed Diabetes, National Health Interview Survey 2016-2017

	Diagnosed Prediabetes†				High Risk, No Diagnosed Prediabetes ‡			
	**Increasing physical activity**	**Reducing the amount of fat or calories in diet**	**Participating in weight loss program**	**Participated in program to prevent Type 2 diabetes**	**Increasing physical activity**	**Reducing the amount of fat or calories in diet**	**Participating in weight loss program**	**Participated in program to prevent Type 2 diabetes**
**Total**	63.2 (61.2, 65.2)	65.6 (63.5, 67.7)	12.7 (11.3, 14.3)	3 (2.3, 3.9)	52.3 (51, 53.7)	52.7 (51.3, 54)	8.4 (7.8, 9)	0.7 (0.5, 0.9)
**Year**								
2016	63.1 (60.2, 65.9)	66.1 (63.1, 69)	11.9 (10.1, 13.8)	2.7 (1.9, 3.9)	50.4 (48.7, 52)	50.9 (49.2, 52.5)	8.1 (7.3, 8.9)	0.6 (0.5, 0.9)
2017	63.4 (60.5, 66.1)	65.2 (62.4, 67.8)	13.5 (11.4, 15.9)	3.3 (2.3, 4.6)	54.3 (52.6, 56.1)	54.6 (52.9, 56.2)	8.7 (7.8, 9.7)	0.7 (0.5, 1)
p-value	0.89	0.62	0.25	0.43	<0.001	<0.001	0.32	0.43
**Age group**								
18-44	64.3 (59.8, 68.6)	61.4 (56.4, 66.1)	11.6 (8.8, 15.1)	1.8 (1, 3.4)	48.7 (44.6, 52.8)	46.1 (42.4, 49.8)	8.6 (6.7, 10.8)	0.6 (0.2, 1.3)
45-64	66.6 (63.4, 69.6)	69.7 (66.5, 72.6)	14.7 (12.5, 17.2)	3.6 (2.6, 5)	54.6 (52.9, 56.4)	55.7 (54, 57.4)	9 (8.1, 10)	0.6 (0.5, 0.9)
65+	56.6 (52.8, 60.3)	63 (59.6, 66.2)	10.6 (8.7, 12.9)	3.4 (2.2, 5.1)	50.1 (48.2, 51.9)	50.3 (48.4, 52.1)	7.4 (6.5, 8.4)	0.8 (0.5, 1.1)
p-value	<0.001	0.002	0.02	0.1	<0.001	<0.001	0.06	0.1
**Sex**								
Female	64.1 (61.3, 66.8)	69 (66.3, 71.6)	13.3 (11.4, 15.4)	2.9 (2.1, 3.9)	53.9 (52.1, 55.8)	56.1 (54.4, 57.9)	9.5 (8.7, 10.5)	0.8 (0.6, 1.1)
Male	62.2 (59, 65.4)	61.6 (58.3, 64.8)	12.1 (10, 14.6)	3.1 (2.1, 4.7)	51.2 (49.5, 52.8)	50.2 (48.4, 51.9)	7.5 (6.7, 8.4)	0.6 (0.4, 0.8)
p-value	0.39	<0.001	0.46	0.79	0.02	<0.001	0.001	0.79
**Race/ethnicity*** **								
NH white	61.5 (59.1, 63.8)	63.2 (60.5, 65.8)	12.4 (10.7, 14.4)	2 (1.4, 2.9)	51.5 (50.1, 52.9)	51.4 (49.9, 52.9)	8.6 (7.8, 9.4)	0.5 (0.4, 0.7)
NH black	66.5 (60.7, 71.7)	68.8 (62.8, 74.2)	10.3 (7.6, 13.8)	5 (3.1, 8.2)	54.9 (51.2, 58.5)	54.7 (51.3, 58)	8 (6.6, 9.7)	1.3 (0.7, 2.5)
NH Asian	71.2 (61.3, 79.4)	70.9 (62.2, 78.2)	18.5 (11, 29.4)	3.5 (1.2, 9.8)	54.6 (47.2, 61.9)	54.1 (47.8, 60.3)	5.9 (3.2, 10.4)	--

© 2019 Ali MK et al. *JAMA Network Open.*

Hispanic	63.7 (57.6, 69.5)	69.9 (64.3, 75)	13.3 (9.1, 19)	6 (3.2, 10.9)	54 (50.1, 57.9)	57.9 (53.2, 62.5)	8 (6.3, 10.2)	0.8 (0.4, 1.5)
p-value	0.20	0.08	0.30	0.007	0.26	0.09	0.73	0.007
**Education**								
<HS	58.5 (52.4, 64.3)	60 (53.3, 66.3)	7.6 (4.5, 12.6)	1.2 (0.6, 2.4)	39.1 (36, 42.2)	38.5 (35.3, 41.8)	3.3 (2.3, 4.6)	0.4 (0.2, 0.7)
HS	57.2 (52.4, 61.8)	58.2 (53.7, 62.6)	10 (7.6, 13)	2.5 (1.5, 4.1)	46.5 (44.3, 48.6)	47.7 (45.6, 49.8)	6.4 (5.5, 7.4)	0.6 (0.4, 1.0)
>HS	66.8 (64.3, 69.2)	69.8 (67.5, 72.1)	15 (13.1, 17.1)	3.8 (2.7, 5.2)	57.8 (56.2, 59.5)	58 (56.4, 59.6)	10.3 (9.5, 11.2)	0.8 (0.6, 1.1)
p-value	<0.001	<0.001	0.001	0.02	<0.001	<0.001	<0.001	0.02
**Insured**								
No	52.9 (44.1, 61.5)	62.9 (53.9, 71.2)	10.4 (6.2, 16.8)	2.3 (0.4, 11.1)	40.9 (36.7, 45.3)	43.2 (38.8, 47.7)	6.4 (4.3, 9.6)	--
Yes	63.9 (61.7, 66)	65.8 (63.6, 67.9)	12.9 (11.3, 14.6)	3.1 (2.4, 3.9)	53.2 (51.8, 54.5)	53.4 (52, 54.7)	8.5 (7.9, 9.2)	0.7 (0.5, 0.9)
p-value	0.02	0.53	0.42	0.74	<0.001	<0.001	0.17	--
**GDM** [‖]								
No	62.9 (60.7, 65)	65.9 (63.6, 68.1)	12.2 (10.7, 13.8)	3 (2.3, 3.8)	52.2 (50.9, 53.5)	52.5 (51.1, 53.8)	8.3 (7.6, 8.9)	0.7 (0.5, 0.9)
Yes	67 (59.1, 74.1)	62.2 (53.4, 70.2)	18.7 (13.7, 25)	3.1 (0.9, 10.4)	57.8 (50.6, 64.6)	60 (53.1, 66.5)	12.5 (8.1, 18.7)	--
p-value	0.32	0.40	0.01	0.95	0.13	0.04	0.07	--
**HTN**								
No	61.3 (58, 64.4)	62.9 (59.6, 66)	11.2 (9.1, 13.6)	2.5 (1.6, 3.9)	48.9 (47.1, 50.8)	48.5 (46.5, 50.5)	8.4 (7.4, 9.4)	0.6 (0.4, 1.0)
Yes	64.7 (62, 67.4)	67.7 (64.9, 70.5)	14 (12, 16.4)	3.4 (2.5, 4.5)	54.9 (53.4, 56.4)	55.8 (54.2, 57.4)	8.4 (7.6, 9.3)	0.7 (0.5, 1.0)
p-value	0.11	0.02	0.09	0.25	<0.001	<0.001	0.94	0.25
**Body mass index**								
Overweight	63.4 (60.1, 66.7)	61.1 (57.6, 64.5)	10.2 (8.4, 12.4)	2.3 (1.5, 3.6)	49.8 (48, 51.6)	47.7 (46.0, 49.4)	5.8 (5.1, 6.6)	0.6 (0.4, 0.9)
Obese	63.1 (60.2, 65.9)	68.7 (66.1, 71.2)	14.5 (12.5, 16.7)	3.5 (2.6, 4.8)	54.4 (52.7, 56)	56.7 (54.9, 58.4)	10.3 (9.4, 11.4)	0.7 (0.5, 1)
p-value	0.88	<0.001	0.004	0.14	<0.001	<0.001	<0.001	0.14

ADA, American Diabetes Association; HS, high school; NH, non-Hispanic.

© 2019 Ali MK et al. *JAMA Network Open.*

All estimates are weighted percentages with 95% confidence intervals in parentheses calculated from multivariable logistic regression, controlling for all other variables. P-values calculated from an adjusted Wald F test for the associations between strata categories and engagement in each specific diabetes risk-reducing activity.

*Defined as body mass index ≥23 kg/m^2 for Asian adults and ≥25 kg/m^2 for all other adults.

† Diagnosed prediabetes defined by respondents' self-reporting physician diagnosis of prediabetes.

‡ High-risk without a diagnosis of prediabetes defined by ADA score greater than or equal to 5.

*** Estimates for adults of other race/ethnicity not shown.

|| Among women only.

¶ Estimates may be unreliable due to relative standard error >30%.

Overweight was defined as body mass index 23.0-29.9 kg/m^2 for Asian adults and 25.0-29.9 kg/m^2 for all other adults; obese was defined as body mass index ≥30.0 kg/m^2

© 2019 Ali MK et al. *JAMA Network Open.*
